# Mechanism of Bisphenol F Affecting Motor System and Motor Activity in Zebrafish

**DOI:** 10.3390/toxics11060477

**Published:** 2023-05-24

**Authors:** Yeonhwa Kim, Seong Soon Kim, Byeong Heon Park, Kyu-Seok Hwang, Myung Ae Bae, Sung-Hee Cho, Suhyun Kim, Hae-Chul Park

**Affiliations:** 1 Zebrafish Translational Medical Research Center, Korea University, Ansan 15588, Republic of Korea; 2 Bio & Drug Discovery Division, Korea Research Institute of Chemical Technology (KRICT), Daejeon 34141, Republic of Korea; 3 Medical Science Research Center, Ansan Hospital, Korea University, Ansan 15588, Republic of Korea; 4 Chemical Analysis Center, Korea Research Institute of Chemical Technology (KRICT), Daejeon 34114, Republic of Korea; 5 Department of Biomedical Sciences, College of Medicine, Korea University, Seoul 04763, Republic of Korea

**Keywords:** zebrafish, bisphenol F, locomotion, motor neuron, myelination, neurochemicals

## Abstract

Bisphenol F (BPF; 4,4′-dihydroxydiphenylmethane) is one of the most frequently used compounds in the manufacture of plastics and epoxy resins. Previous studies have demonstrated that BPF affects locomotor behavior, oxidative stress, and neurodevelopment in zebrafish. However, its neurotoxic effects are controversial, and the underlying mechanisms are unclear. In order to determine whether BPF affects the motor system, we exposed zebrafish embryos to BPF and assessed behavioral, histological, and neurochemical changes. Spontaneous locomotor behavior and startle response were significantly decreased in BPF-treated zebrafish larvae compared with control larvae. BPF induced motor degeneration and myelination defects in zebrafish larvae. In addition, embryonic exposure to BPF resulted in altered metabolic profiles of neurochemicals, including neurotransmitters and neurosteroids, which may impact locomotion and motor function. In conclusion, exposure to BPF has the potential to affect survival, motor axon length, locomotor activity, myelination, and neurochemical levels of zebrafish larvae.

## 1. Introduction

Bisphenol A (BPA) is the most commonly used material in the manufacture of polycarbonate and epoxy resins used for the production of plastic bottles, plastic food containers, medical devices, and thermal paper [1]. However, numerous studies have shown a relationship between BPA toxicity and diseases in humans, such as cardiovascular disease, diabetes, developmental disorders, reproductive disorders, cancer, and chronic kidney and respiratory disease [2].

Several BPA analogs, such as bisphenol B (BPB), bisphenol B (BPF), and bisphenol S (BPS), have been developed and used as alternatives to avoid BPA toxicity. In particular, BPF is used as a substitute for BPA in items such as household products and packaging materials. BPA analogs cause oxidative stress both in vivo and in vitro in rat models [3] and have similar estrogenic effects in fish [4,5]. In addition, exposure to BPF and BPS leads to reproductive toxicity, immunotoxicity, and oxidative stress, as well as endocrine-disrupting activity in 3T3-L1 cells [6]. BPF has also been shown to alter spermatogenesis in the testes and reduce testosterone secretion in male rats [7]. These results indicate that replacing BPA with BPA analogs is also unsafe.

The zebrafish is a valuable model for assessing the toxicity of various chemicals [8] because of several advantages, including its high similarity with human genetics and organs, high reproductive capacity, and external fertilization and development. The optical transparency of zebrafish embryos allows for the direct and continuous visualization of morphogenesis under the microscope. In particular, zebrafish and humans share many neurodevelopmental processes and physiological functions. Owing to these properties, zebrafish models have been widely used to evaluate the effects of developmental neurotoxicity of chemicals.

Acute BPA exposure has been shown to impair numerous neurochemical pathways, including dopaminergic and cholinergic systems, in zebrafish [9]. BPA has also been shown to alter several neurochemicals, including cholinergic, serotonergic, kynurenergic, dopaminergic, and GABAergic components in zebrafish larvae [9]. A recent study demonstrated that exposure to BPF (70 and 700 µg/L) decreases locomotor behavior, increases oxidative stress, and delays neurodevelopment in zebrafish [10]. Exposure to 0.5 and 5.0 mg/L BPF caused a defect in the spinal cord and inhibited spontaneous movements in zebrafish [11]. Despite a large number of such studies, it remains unclear whether BPF affects motor systems and neurochemicals. Given the advantages of the zebrafish model system, we evaluated BPF neurotoxicity using zebrafish larvae. We assessed the effects of BPF exposure on behavior and neurotransmitter levels in zebrafish larvae to verify whether exposure to BPF affects the motor nervous system during the developmental process.

## 2. Materials and Methods

### 2.1. Zebrafish Lines

Wild-type and Tg(Hb9:GFP) [12], Tg(Olig2:dsRed) [13], Tg(Hb9:mCherry) [14], Tg(Mpb:EGFP) [15], and Tg(Claudink:gal4;5UAS:mGFP) [16] zebrafish were used in this study. Zebrafish were kept in an autosystem at 28.5 °C with a 14:10 h light:dark cycle in an aquarium container and were fed twice daily with brine shrimp. For imaging, zebrafish embryos were kept in an incubator at 28.5 °C. For behavioral tests, zebrafish embryos were kept in an aquarium. Zebrafish embryos were staged according to the days post-fertilization (dpf).

### 2.2. Chemicals

BPF (cat. no. 51453), BPA-d_16_ (cat. no. 451835), 3,4-dichloroaniline (3,4-DCA, cat. no. 437778), Dimethyl sulfoxide (DMSO, cat. no. 472301), and tricaine (MS-222) were purchased from Sigma-Aldrich (St. Louis, MO, USA). 1-Phenyl-2-thiourea (PTU) was purchased from Fluka (CAS No. 79180).

Standards for the three neurosteroids (progesterone, allopregnanolone, and testosterone) and six neurotransmitters (3-methoxytyramine, 3,4-dihydroxy-L-phenylalanine, norepinephrine, glutamic acid, gamma-aminobutyric acid, and dopamine) used in this study were purchased from either Sigma-Aldrich (St. Louis, MO, USA) or Steraloids, Inc. (Newport, RI, USA). The following internal standards were used: dehydroepiandrosterone-2,2,3,4,4,5-d6 for testosterone; pregnenolone-17β,21,21,21-d4 for progesterone and allopregnanolone; and tryptophan-d3 for the aforementioned six neurotransmitters; these standards were purchased from Sigma-Aldrich and C/D/N Isotopes Inc. (Pointe-Claire, QC, Canada). All organic solvents were of analytical or HPLC grade and purchased from Honeywell Burdick and Jackson (Muskegon, MI, USA). Deionized water was prepared using a Milli-Q purification system (Millipore, Burlington, MA, USA).

### 2.3. EC/LC Test

Following the OECD guidelines (No. 236), we established controls, including a negative control (E3 media, 15 mM NaCl, 0.5 mM KCl, 1 mM CaCl_2_, 1 mM MgSO_4_, 0.15 mM KH_2_PO_4_, 0.05 mM NH_2_PO_4_, and 0.7 mM NaHCO_3_), a positive control (3,4-DCA), and a solvent control (DMSO). Wild-type zebrafish embryos at 4 h post-fertilization (hpf) were exposed to a series of BPF doses (1, 2.2, 4.6, 10, 22, 46, and 100 mg/L), 3,4-DCA (4 mg/L), and 0.1% DMSO in E3 embryo medium with an exposure time of up to 120 hpf. A total of 20 embryos were exposed to each dose in a 96-well plate (SPL, cat no. 34296), with one embryo per well. At 4 hpf, embryos developing normally at the blastula stage (30% epiboly) were sorted using a microscope and subsequently used for further experiments. Zebrafish embryo survival was examined in terms of hatching, malformation, and lethality at 24, 48, 72, 96, and 120 hpf. Mortality was measured on the basis of missing heartbeats. Malformation was defined as heart malformation, pericardial edema, uninflated swim bladder, and yolk sac edema at 120 hpf. Ten embryos, randomly selected from each experimental group, were used to obtain morphological images under a microscope. Experiments were performed in triplicate for each concentration. The LC_20_ and EC_20_ values were calculated using the Excel program (Microsoft). After drawing the graph with the value calculated as a percentage, the concentration values were inserted into the formula generated by inserting the trend line of the graph. (LC_20_, y = −0.0236x^2^ + 3.0706x−33.034, R^2^ = 0.8576; EC_20_, y = −0.0651x^2^ + 6.114x−3.2135, R^2^ = 0.997).

### 2.4. BPF Uptake Concentration

Wild-type zebrafish embryos at 4 hpf were exposed to BPF doses of 5 and 10 mg/L in E3 embryo medium for an exposure time of up to 120 hpf. For the BPF uptake concentration analysis, samples from 30 zebrafish larvae were extracted according to previously described methods [17]. BPA-d_16_ was used as the internal standard.

### 2.5. Transgenic Zebrafish Live Imaging

Transgenic zebrafish embryos at 4 hpf were exposed to a series of BPF doses of 1, 5, and 10 mg/L, and 0.1% DMSO in 0.003% (*w*/*v*) PTU solution for up to 72 hpf or 120 hpf, depending on the type of test. In all experiments, 20 embryos were exposed to each dose in a 6-well plate (SPL, cat. no. 30006) with 20 embryos per well. All the experimental larvae expressing dsRed or GFP were selected under a fluorescence microscope (SMZ18, Nikon, Tokyo, Japan). All fluorescence images were obtained using a spinning confocal microscope (CSU-X1, Nikon, Tokyo, Japan). The length of motor axons and myelin sheath, and the number of neurons and oligodendrocytes were measured using the cell counter and length measurements of NIS-Elements AR Analysis 4.30 software (Nikon, Tokyo, Japan).

### 2.6. Terminal Deoxynucleotidyl Transferase dUTP Nick End Labeling (TUNEL) Staining and Immunohistochemistry

Embryos were fixed using 4% paraformaldehyde and embedded in 1.5% agar blocks containing 5% sucrose and equilibrated in 30% sucrose solution. The frozen blocks were cut into 10 µm sections using a cryostat microtome (CM1860, Leica, Nussloch, Germany) and mounted on glass slides. The sections were rinsed three times with 1× phosphate-buffered saline (PBS), treated with permeabilized solution (10 mM citric acid with 1% Triton-X) at 4 °C for 2 min, and washed twice with PBS. The TUNEL mixture (Roche) was incubated in a humidified incubator at 37 °C for 60 min. The sections were blocked with PBS containing 2.5% sheep serum and 2.5% bovine serum albumin (BSA) at RT for 60 min and incubated overnight at 4 °C with primary antibody (chick anti-GFP, Abcam, 1:200). The sections were then rinsed 10 times with 1× PBS, blocked with PBS containing 2.5% sheep serum and 2.5% BSA at RT for 1 h, and incubated overnight at 4 °C with secondary antibody (Flour 488-conjugated, Molecular Probe, 1:1000). The sections were then rinsed thrice with 1× PBS. The nuclei were stained with 4′,6-diamidino-2-phenylindole, dihydrochloride (DAPI) (D1306, Thermo Fisher Scientific). Finally, the sections were washed several times with PBS and mounted. All the larvae were imaged using a confocal microscope (A1si, Nikon) equipped with a Nikon Plan Apo 40×/1.25 objective.

### 2.7. Behavior Analysis

Wild-type zebrafish embryos at 4 hpf were exposed to a series of BPF doses (1, 5, and 10 mg/L) and 0.1% DMSO in E3 embryo medium for an exposure time of up to 120 hpf. A total of 60 embryos were exposed to each dose in a Petri dish (SPL, cat. no. 20101). Larvae (120 hpf) were placed in 48-well plates (SPL, cat. no. 30048), with one larva per well containing 500 µL of treatment solution in each experimental group. For adaptation, the plates were placed in a behavior test room for 2 h and subsequently placed in a DanioVision Observation Chamber (Noldus, Wageningen, The Netherlands). The light/dark behavior test consisted of first light state for 20 min, followed by two cycles of dark state for 5 min and light state for 5 min, and lastly, 20 min of dark state. The video recordings were analyzed using the EthoVision XT program (Noldus). Locomotor activity was measured using mean velocity (mm/s). To analyze the startle response, 60 embryos were exposed to each dose in a Petri dish (SPL, cat no. 20101). Larvae (120 hpf) were placed in 48 well plates (SPL, cat no. 30048), with one larva per well containing 500 µL of treatment solution in each experimental group. The startle was measured using the mechanically tapping stimulation via the solenoid of the DanioVision chamber. Stimulus intensity was set at level 3 in the EthoVision XT program. The difference between the response when no stimulus was given (spontaneous response) and the response when stimulated (startle response) was compared and analyzed by distance moved (mm), latency to startle (seconds), and peak distance moved (mm). All recordings were obtained from the DanioVision’s built-in camera shooting at 30 frames per second.

### 2.8. Neurochemical Analysis

Samples from the 25 zebrafish larvae were pooled to form a composite sample. For neurotransmitter analysis, samples were extracted according to a previously reported method [9]. Briefly, zebrafish larvae were lysed by snap-freezing three times using liquid nitrogen. Then, 100 µL of distilled water containing 0.1% formic acid was added to the lysed zebrafish, and the samples were homogenized using an ultra sonicator (VCX-130, Sonics & Materials Inc., Newtown, CT, USA). Then, the endogenous neurotransmitters were extracted by adding 100 µL of MeOH containing 1% formic acid, followed by vortexing and centrifugation at 21,130× *g* for 10 min. The supernatants were analyzed using liquid chromatography-mass spectrometry (LC-MS/MS) analysis

For the neurosteroid analysis, the samples were prepared as described previously with a few modifications [18]. First, 1 mL of MeOH/acetic acid (99:1 *v*/*v*) containing internal standards was added to the samples and vigorously homogenized, followed by extraction at 4 °C for 24 h. Next, the samples were centrifuged at 21,130× *g* for 5 min, and the pellet was further extracted twice with 1 mL of MeOH/acetic acid (99:1 *v*/*v*). The organic phases were collected and dried. The remaining samples were resuspended in 1 mL of MeOH/water (10:90, *v*/*v*), and the mixture was extracted using SPE cartridges (Waters^TM^ Oasis PRiME HLB, Newberg, OR, USA) under vacuum conditions according to the manufacturer’s guidelines. The final eluates were dried under a stream of nitrogen and dissolved in 100 µL of methanol. Finally, the clear solution was injected into the LC-MS/MS.

### 2.9. Statistical Analysis

Graph preparation and statistical analyses were performed using GraphPad Prism 9 (GraphPad Software, Inc., La Jolla, CA, USA). Two-way analyses of variance were performed when comparing the experimental groups to analyze locomotor responses to BPF exposure. Post hoc analysis was performed using Bonferroni’s multiple comparisons test. One-way analyses of variance were performed when comparing the experimental groups to analyze the startle response, neurotoxic effects and neurotransmitter activities after BFP exposure. Post hoc analysis was performed using Tukey’s method. The *p*-values obtained from two-way analyses of variance and one-way analyses of variance were adjusted by Bonferroni correction. For the neurotransmitter analysis, data manipulation was performed using Excel 2013 (Microsoft Corporation, Seattle, WA, USA) and GraphPad Prism 9. The groups were compared using an unpaired two-tailed Student’s t-test for the BPF uptake. All data are expressed as means ± SD. * *p* < 0.05, ** *p* < 0.01, and *** *p* < 0.005 were considered statistically significant.

## 3. Results

### 3.1. Determination of Lethal and Effective Concentration (LC_20_, EC_20_) Values of BPF for Zebrafish Larvae

**Figure 1:**
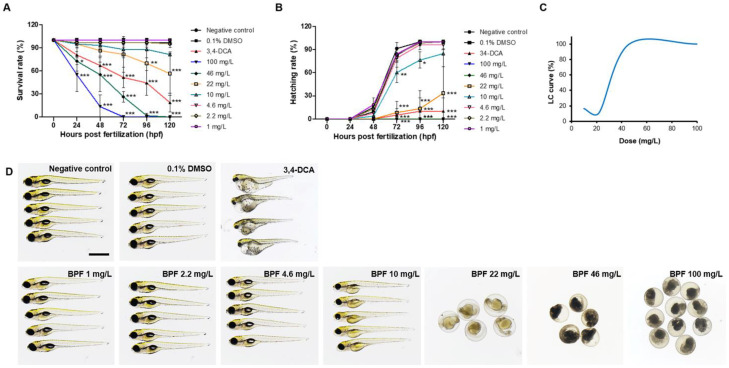
Toxicity of BPF on mortality and hatching rate of zebrafish embryos and larvae. (**A**) Mortality of zebrafish exposed to BPF and control at 120 hpf. (**B**) Hatching rate of zebrafish exposed to BPF and control at 72 hpf. No embryos in the 22 mg/L exposure group hatched. (**C**) Representative LC curve graph of BPF. (**D**) Morphology of zebrafish larvae at 120 hpf exposed to BPF doses. *n* = 16 in each concentration; scale bar, 500 µm. * *p* < 0.05; ** *p* < 0.01; *** *p* < 0.001.

To verify the effect of BPF on embryonic development, we confirmed the morphological changes in zebrafish exposed to BPF and analyzed its effects on developmental toxicity. Survival rate, hatching rate, and morphological abnormalities were measured to determine the developmental toxicity of BPF. Embryos were exposed to a dose of BPF, and the calculated LC_20_ and EC_20_ values of BPF were 12 mg/L (120 hpf) and 4 mg/L (120 hpf), respectively (Figure 1A,C). Zebrafish embryos exposed to BPF showed a dose-dependent delay in hatching rate (Figure 1B). At high concentrations (46 mg/L and 100 mg/L), all embryos died within 96 hpf. In addition, 22 mg/L BPF induced yolk sac edema, pericardial edema, and delayed hatching. Swim bladders did not form in zebrafish treated with 10 mg/L BPF (Figure 1D). The concentration of 5 mg/L, which was similar to the EC_20_ value at which the zebrafish embryos did not show morphological abnormalities, and the concentration of 10 mg/L, which was similar to the LC_20_ value, were selected for the neurotoxicity test. These data revealed that BPF induced developmental defects in zebrafish embryos, including hatching inhibition, depigmentation, yolk sac edema, and pericardial edema.

### 3.2. Accumulation of BPF in the Zebrafish Larvae Body Increased in a Concentration-Dependent Manner

**Figure 2:**
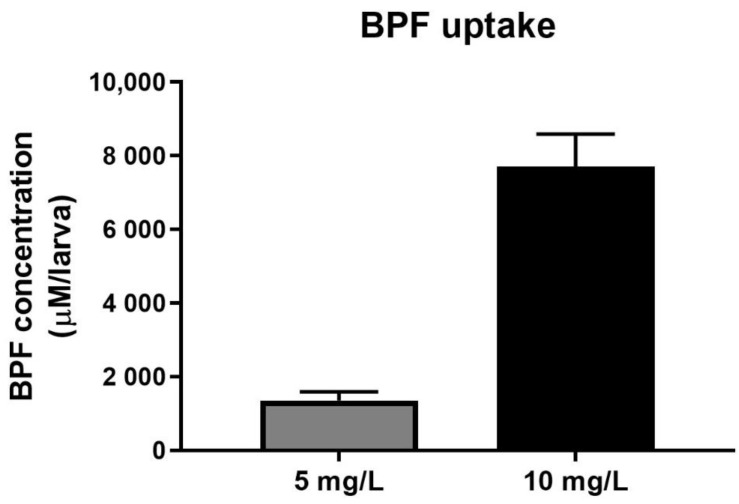
Measurement of BPF concentration in the zebrafish larvae. The concentration of BPF entering the body of zebrafish larvae exposed to 5 mg/L and 10 mg/L BPF for 5 days was measured. *n* = 30 for each concentration.

To confirm the accumulation of BPF in the body, we evaluated the accumulation of BPF in zebrafish larvae exposed to water containing the selected concentrations of BPF. The measured BPF concentration per zebrafish larva exposed to 5 mg/L and 10 mg/L BPF was 1358.1 ± 233.2 µM and 7705.4 ± 881.9 µM, respectively (Figure 2). The BPF concentration increased by nearly four-fold in zebrafish larvae exposed to 10 mg/L BPF compared with larvae exposed to 5 mg/L BPF. These results indicate that BPF is highly absorbed into zebrafish and accumulates in the body, depending on the exposure concentration.

### 3.3. BPF Impaired the Locomotor Activity in Zebrafish Larvae

**Figure 3:**
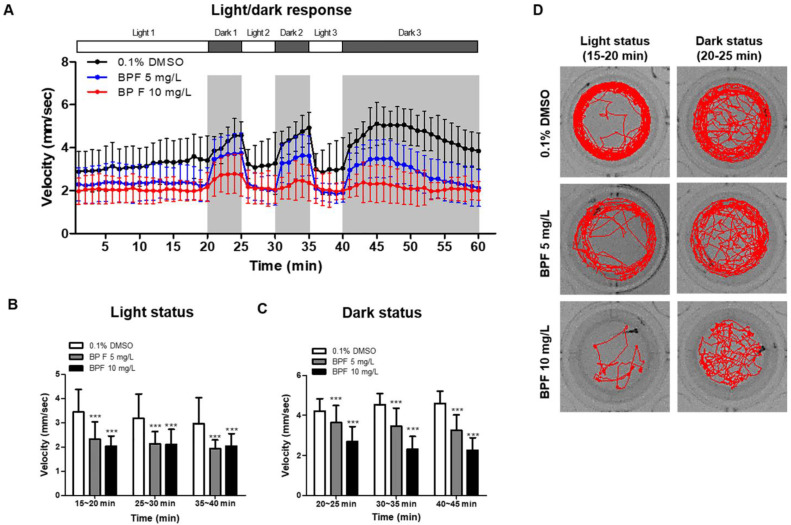
Light-dark behavior test in BPF-exposed zebrafish larvae. (**A**) Zebrafish larvae at 5 dpf were adapted in light status for 20 min, and subjected to two cycles of 5 min of light/5 min of dark periods. (**B**,**C**) Velocity in light status and dark status. (**D**) Representative images of the transition for 5 min each in light and dark status. *n* = 48 in each concentration. Data are presented as means ± SD. *** *p* < 0.001.

To determine the effects of BPF exposure on locomotor activity in alternating periods of light and dark (Figure 3A), zebrafish embryos (4 hpf) were exposed to BPF (5 mg/L and 10 mg/L) until 5 dpf. Zebrafish larvae were individually placed in 48-well plates with embryo E3 medium containing 5 mg/L or 10 mg/L BPF. The velocity was significantly decreased in BPF (5 mg/L and 10 mg/L)-treated zebrafish larvae compared with that in the control (Figure 3B,D). In addition, the velocity was significantly decreased in BPF-exposed zebrafish larvae compared with that in the DMSO control (*p* < 0.001) in the dark (Figure 3C,D). In the dark, BPF-exposed larvae showed not only decreased locomotor activity but also decreased preference for the outer zone (thigmotaxis) (Figure S1). These data suggest that BPF exposure during early embryonic development impairs locomotor behavior in zebrafish.

### 3.4. BPF Reduced Startle Response in Zebrafish Larvae

**Figure 4:**
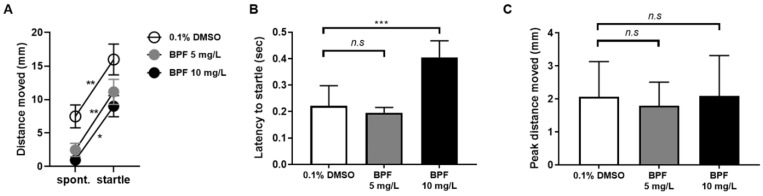
Characterization of the startle response of 5 dpf zebrafish larvae. (**A**) The distance moved was compared between spontaneous response and startle response after stimulus within each group. (**B**) The peak distance moved in response to the stimulus was compared immediately after the startle stimulus was provided. (**C**) Latency to startle time after stimulation was compared with that of the DMSO control group. *n* = 26 for DMSO control and BPF 5 mg/L, *n* = 13 for BPF 10 mg/L. Data are presented as means ± SD. n.s.—not significant, * *p* < 0.05; ** *p* < 0.01; *** *p* < 0.001.

In addition to locomotor behavior, we investigated whether BPF exposure affected the startle response to external stimuli. To reveal the effect of BPF exposure on startle response to the tapping stimulus, we characterized the startle response by the total distance moved by the zebrafish larvae, latency to startle, and peak distance moved after tapping stimulation. The response to tapping stimulus was similar in both the control and BPF-exposed groups, even though the basal level of locomotor activity was low in the BPF-treated group (Figure 4A). The values for the peak distance moved did not change in the 10 mg/L BPF-treated group (Figure 4B); however, a significant increase in the latency to startle response was observed in this group compared with the control group (Figure 4C). These results indicate that BPF exposure induced a delayed response to stimuli as well as locomotor activity.

### 3.5. Effects of BPF Exposure on Motor System

**Figure 5:**
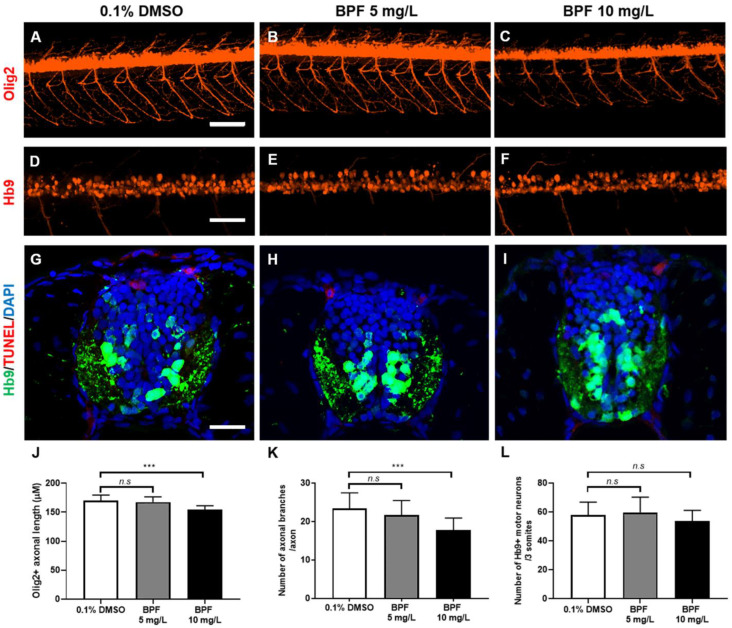
Toxic effects of BPF on motor axons and neurons of zebrafish larvae at 3 dpf. (**A**–**C**) Lateral views of the spinal cord of Tg(Olig2:dsRed) at 3 dpf. (**D**–**F**) Lateral views of the spinal cord of Tg(Hb9:mCherry) at 3 dpf. The motor neurons and interneurons expressed mCherry fluorescence. (**G**–**I**) Transverse section images of spinal cord of Tg(Hb9: GFP) zebrafish. The dead neurons (red spots) were confirmed through TUNEL assay. (**J**) The graph represents the length of olig2 positive motor axons. *n* = 12 for each concentration. (**K**) The graph represents the number of axonal branches per motor axon. *n* = 12 for each concentration. (**L**) The graph represents the number of Hb9 positive motor neurons. *n* = 15 for each concentration. Data are presented as means ± SD. n.s.—not significant, *** *p* < 0.001. (**A**–**C**) Scale bar, 100 µm. (**D**–**F**) Scale bar, 50 µm. (**G**–**I**) Scale bar, 20 µm.

Since locomotor behavior was impaired upon BPF exposure, we investigated whether BPF exposure altered the formation of motor systems, including motor neurons, axons, and skeletal muscles. The motor axonal length, measured from the sagittal plane, accurately represented the length of the 3D axonal trajectory along the Z-axis. Motor axonal length was significantly reduced in zebrafish larvae treated with 10 mg/L BPF compared with the solvent control larvae (Figure 5A–C,J). Furthermore, the number of motor axon branches was reduced without alterations in neuromuscular junctions in BPF-treated zebrafish (Figure 5K and Figure S2). BPF exposure did not cause changes in the actin cytoskeleton of skeletal muscles (Figure S3), indicating that impaired locomotion was caused by neurotoxicity. To identify whether BPF induced the degeneration of motor neurons, we observed the formation of motor neurons and cell death. Because the Hb9 promoter drives the expression of fluorescent receptors in motor neurons and interneuron subsets [19,20], we used Tg(Hb9;mCherry) to observe the formation of motor neurons. The number of motor neurons and interneuron subsets did not change in the BPF-treated zebrafish larvae (Figure 5D–F,L). In addition, the number of TUNEL+ cell deaths in the larval spinal cord did not differ between the 0.1% DMSO control and BPF-treated groups (Figure 5G–I). These results indicate that BPF–exposed larvae showed the degeneration of motor axons without causing the death of motor neurons.

### 3.6. BPF Affected the Myelination of Zebrafish Larvae

**Figure 6:**
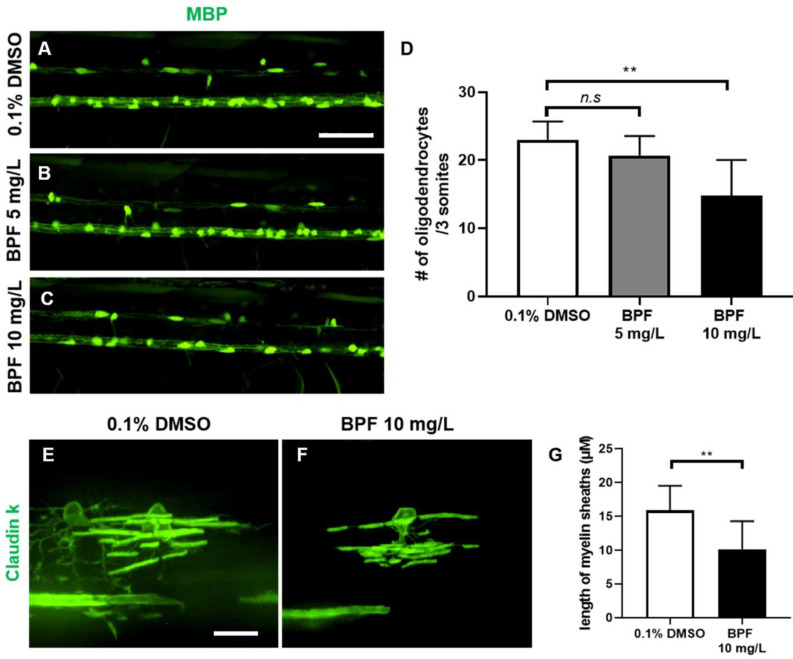
Toxic effects of BPF on oligodendrocytes of zebrafish larvae at 5 dpf. (**A**–**C**) Lateral views of the spinal cord of Tg(MBP:EGFP) at 5 dpf. The oligodendrocytes expressed EGFP fluorescence. (**D**) The graph compares the number of oligodendrocytes per three somites in 0.1% DMSO and 5, 10 mg/L BPF-treated zebrafish. *n* = 10 for DMSO control and BPF 10 mg/L, *n* = 13 for BPF 5 mg/L. (**E**,**F**) The myelin sheaths were expressed in GFP in Tg(ClaudinK:mGFP). (**G**) The graph compares the average length of myelin sheaths per oligodendrocyte in 0.1% DMSO and zebrafish treated with 10 mg/L BPF. *n* = 15 for DMSO control, *n* = 10 for BPF 10 mg/L. Data are presented as means ± SD. n.s.—not significant, ** *p* < 0.01. (**A**–**C**) Scale bar, 100 µm. (**E**–**F**) Scale bar, 50 µm.

Myelination is important for proper motor function. Therefore, to investigate the impact of BPF on myelination, we observed the formation of oligodendrocytes that produce myelin sheaths and synthesize myelin. The number of oligodendrocytes did not decrease in zebrafish larvae treated with 5 mg/L BPF. However, the number of oligodendrocytes was significantly decreased in zebrafish larvae treated with 10 mg/L compared with the control group (Figure 6A–D). Furthermore, the average length of the myelin sheath produced per oligodendrocyte was greatly decreased in BPF-treated zebrafish (Figure 6E–G). These results indicate that BPF exposure disrupts oligodendrocyte formation and myelin synthesis.

### 3.7. BPF Exposure Altered Metabolic Neurochemicals in Zebrafish Larvae

**Figure 7:**
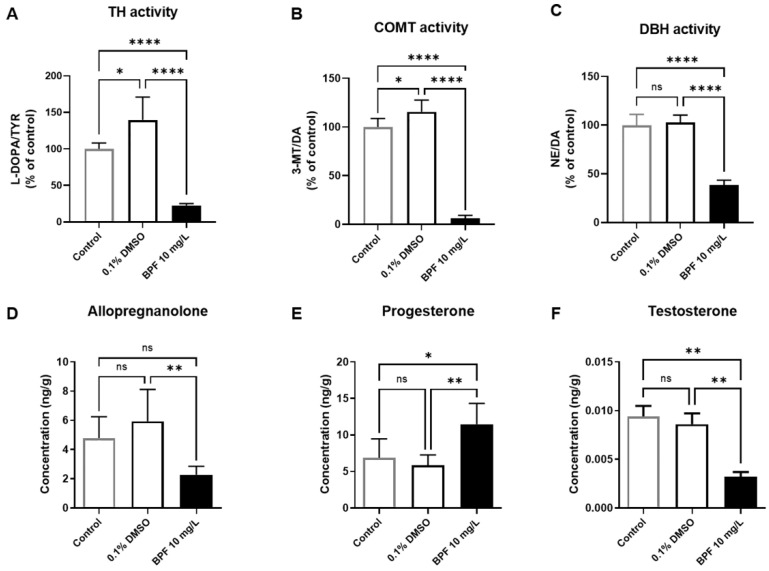
Effects of BPF on neurochemicals in zebrafish larvae at 5 dpf (*n* = 25 pooled; five replicates). Enzymatic activities of TH (**A**), COMT (**B**), DBH (**C**), and the levels of allopregnanolone (**D**), progesterone (**E**), and testosterone (**F**) in zebrafish. Data are presented as means ± SD. The sample from 25 individual zebrafish larvae was pooled to form a composite sample. n.s.—not significant, * *p* < 0.05; ** *p* < 0.01; **** *p* < 0.0001.

To investigate alterations in neurochemicals and neurotoxicity induced by BPF exposure in zebrafish, we quantitatively analyzed neurotransmitters and neurosteroids using LC-MS/MS. In zebrafish larvae exposed to BPF, we observed significant decreases in the enzymatic activities of tyrosine hydroxylase (TH), catechol-O-methyltransferase (COMT), and dopamine beta-hydroxylase (DBH) compared with the controls treated with 0.1% DMSO (Figure 7A–C). Additionally, we found that the levels of allopregnanolone and testosterone significantly decreased, whereas those of progesterone significantly increased in the BPF-exposed group compared with the controls treated with 0.1% DMSO (Figure 7D–F). TH and COMT activity differed slightly between the untreated control group and the group treated with 0.1% DMSO, whereas the levels of other neurochemicals were not different between the two groups.

## 4. Discussion

Numerous studies have demonstrated that BPA affects human health, impacting reproduction, development, metabolic diseases, and the immune system [1]. However, research on the effects of BPF on human health is much less common than those of BPA. A recent study showed that BPA affects obesity in children and adolescents in the United States [21]. Previous studies have focused on reproductive functions and antioxidant systems [7,22]. Exposure to BPF leads to increased oxidative stress and reduced expression of neurodevelopment-related genes [10]. In this study, we focused on the effects of BPF on the motor system and locomotor activity.

BPA-treated zebrafish larvae exhibited hypolocomotion in a dose-dependent manner compared with those in the control group [9,23,24]. Another study showed that BPA (0.01, 0.1, or 1 μM) led to hyperactivity in zebrafish larvae [25]. These results suggest that the behavioral changes in zebrafish larvae vary according to the dose of BPA. In addition, startle response upon touch stimuli or swim speed in spontaneous locomotor activity were significantly decreased in 15 μM BPA-treated zebrafish larvae [23], similar to our results. Although the species is different from zebrafish, BPA-exposed rats also exhibited increased acoustic startle reflex and latency to groom [26]. These results indicated that BPA causes an altered response to stimuli. We found that BPF affected locomotor activity and startle responses to stimuli in zebrafish larvae. However, exposure to 100 μg/L BPF did not decrease the distance moved [24]. Together with the results of previous studies, these results suggest that behavioral alterations manifest differently following concentration-modulated BPF exposure, compared with those following BPA exposure. These findings suggest that BPF could induce locomotion impairment similar to that induced by BPA. In addition, our results showed that thigmotaxis was reduced in BPF-treated zebrafish larvae (Figure S1). A previous study suggested that behaviors indicating anxiety and depression were increased in BPF-exposed rat offspring [27]. These results suggested that BPF has potential to affect emotion, including anxiety, and further studies should be performed to reveal the effect of BPF.

Reduced motor axon length is related to increased motor neuron cell death rate [28]. Therefore, measuring motor axonal length is a common method for estimating motor axonal degeneration. Our results indicate that the axon length of motor neurons was significantly decreased in BPF-exposed zebrafish. This finding is similar to that of a previous study that revealed that 0.5 and 5 mg/L BPF significantly inhibited motor axon growth in Mn-GF transgenic zebrafish at 72 hpf [11]. In addition, a previous study showed that BPA exposure reduces motor axonal length and branching in zebrafish embryos at 48 hpf [28]. Therefore, similar to BPA, BPF exposure affects motor neurons during the neurodevelopmental processes. However, BPF did not affect the number or apoptosis of motor neurons at the exposed concentration. This suggests that BPF exposure in our analysis only induced axonal degeneration prior to cell degeneration and indicates the possibility that an increase in exposure duration or concentration may lead to cell degeneration.

BPA exposure alters myelination in the hippocampus of developing rat brains [29]. Furthermore, BPA reduces the number of oligodendrocytes in rat pups and decreases *mbp* gene expression levels in the hippocampus of adult rats [30]. The number of immature oligodendrocytes increased in BPA- and BPF-treated rat fetal neural stem cells [31]. In this study, we demonstrated that the numbers of oligodendrocytes and myelin sheaths decreased in zebrafish larvae exposed to BPF compared with those in the control. This means that BPF exposure during the developmental process affects oligodendrocyte development and myelin synthesis, similar to BPA. Defects in the myelin sheath affect motor control by decreasing the efficiency of neural electrical transmission [32]. This suggests that defects in myelin caused by BPF exposure may lead to a decrease in locomotor function.

Dopaminergic neurons control locomotion through their ascending projections towards the basal ganglia, which project to brainstem locomotor networks. Increased dopamine levels are associated with increased locomotor activity [33,34]. Alterations in TH, COMT, and DBH activities in zebrafish larvae exposed to BPF may affect dopamine-modulated locomotion. The γ-aminobutyric acid A (GABA_A_) receptor is a typical inhibitory neurotransmitter receptor in the central nervous system. GABA signaling affects locomotion and motor functions [35,36,37]. Allopregnanolone is believed to exert a positive modulation on GABA_A_ receptors by binding to specific sites located on both the α subunit and the α/β subunit interface [38]. Upon exposure to BPF, although the levels of progesterone increased, the levels of allopregnanolone (Figure 6E, *p* < 0.05)—which directly modulates the GABA_A_ receptor—decreased. Progesterone is a precursor of allopregnanolone, and its concentration may have increased because exposure to BPF inhibited allopregnanolone metabolism. These results suggest that exposure to BPF disrupts the GABAergic system in zebrafish larvae and reduces locomotion and motor functions. Although testosterone acts as a neurosteroid in the brain, it is produced in various endocrine organs of the zebrafish body and is related to locomotion [39]. The levels of testosterone were significantly decreased in the entire body of zebrafish larvae treated with BPF in comparison with those in the controls. These results suggest that exposure to BPF may decrease locomotor activity by decreasing testosterone levels.

In this study, we confirmed that the decreased motor axon length and locomotor activity induced by BPF in zebrafish larvae is associated with changes in neurochemicals, including the GABAergic and dopaminergic systems. Early exposure to BPA affects dopaminergic and serotonergic systems in juvenile and adult rats [40]. Locomotor activity was affected by increased dopaminergic signaling, including for transporters and receptors, in paraquat-treated zebrafish larvae [41]. BPA, BPS, and BPF significantly alter dopaminergic and serotonergic-related genes in juvenile female rats [42]. Our results have confirmed that BPF, as well as BPA, alters neurochemicals in zebrafish larvae, and this may lead to locomotion-related changes and degeneration of the motor system. BPF concentrations measured in the environment ranged from 1 μg/L to 3 μg/L [43], and the concentrations used in our study are much higher than the environmental level. However, the effects of BPF may be caused by accumulation of toxicity with continuous exposure during a lifetime. Therefore, it is valuable to elucidate the toxicity mechanism of BPF even though the tested concentrations differ from environmental concentrations.

## 5. Conclusions

Our results reveal that exposure to BPF impaired survival, morphological development, motor axon length, locomotor activity, startle response, and myelination and resulted in neurochemical changes in zebrafish larvae. Thus, we demonstrate the toxicity potential of BPF by showing that embryonic exposure to BPF induces neurotoxicity that affects motor function. Further studies are needed to verify the safety of BPA alternatives, including BPF, for human health.

## Data Availability

We have full control of all primary data, and we agree to allow the journal to review our data if requested.

## Supplementary Materials

The following supporting information can be downloaded at: https://www.mdpi.com/article/10.3390/toxics11060477/s1, Figure S1: Effects of BPF on anxiety of zebrafish larvae; Figure S2: Effects of BPF on neuromuscular junction of zebrafish larvae; Figure S3: Effects of BPF on skeletal muscle of zebrafish larvae.
