# Supplementary material for: Mechanism of Bisphenol F Affecting Motor System and Motor Activity in Zebrafish
Source: Toxics. 2023 May 24;11(6):477. doi: 10.3390/toxics11060477 (PMC10302713; doi:10.3390/toxics11060477)
Supplement: Supplementary file 1 [file toxics-11-00477-s001.zip › toxics-2328240-supplementary.pdf]

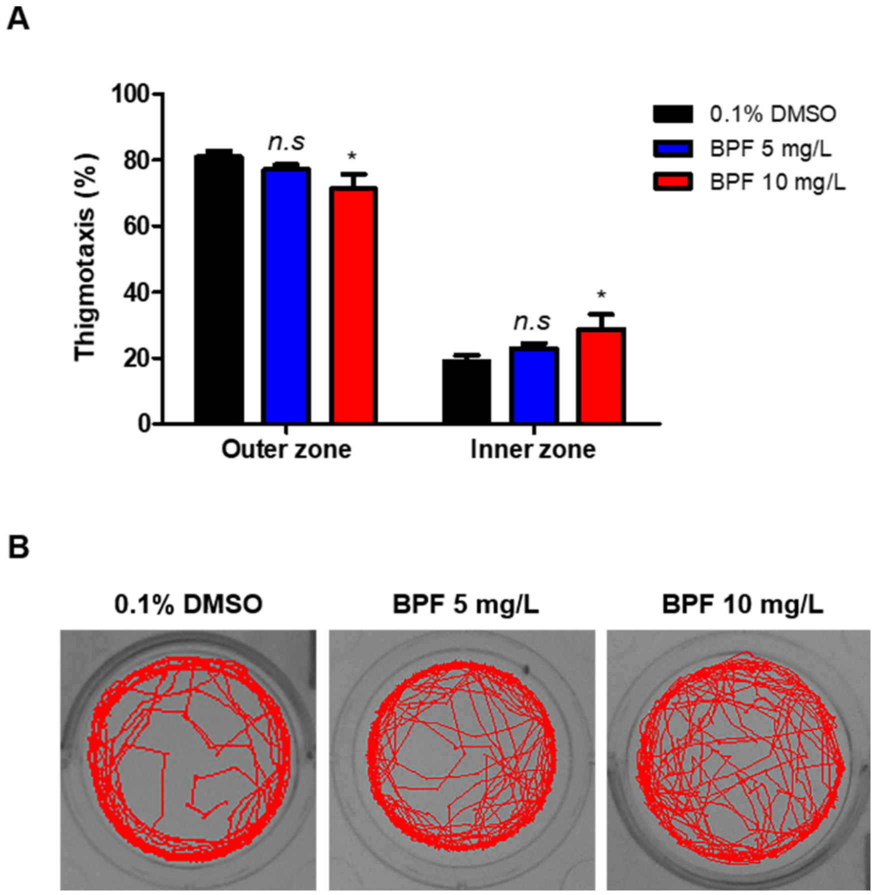

Figure S1. Effects of BPF on anxiety of zebrafish larvae. (A) The thigmotaxis(%) in dark status for 5 min. (B) Representative of the transition for 5 min in dark status. n = 48 in each concentration. Data are presented as means ± SEM. **p* < 0.05.

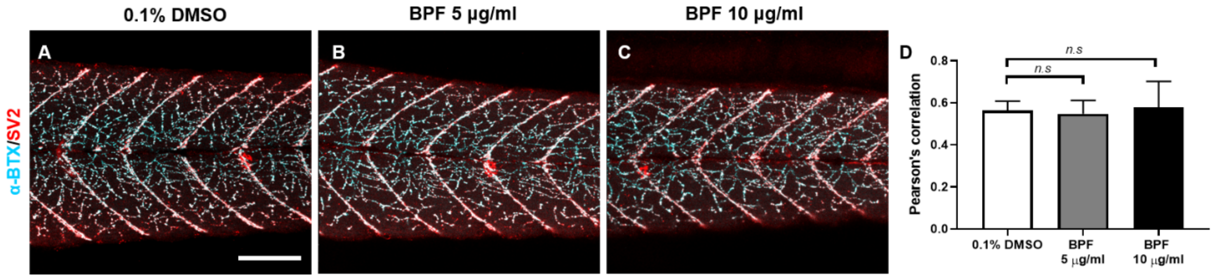

Figure S2. Effects of BPF on neuromuscular junction of zebrafish larvae. (A–C) Lateral views of the spinal cord of zebrafish at 5 dpf. IHC labeling with anti-SV2 antibody (red) and *α*-bungarotoxin (blue). Scale bar, 100 μm. (D) The graph represents quantification of NMJ co-localization. *n.s*; not significant. n = 11 in each concentration.

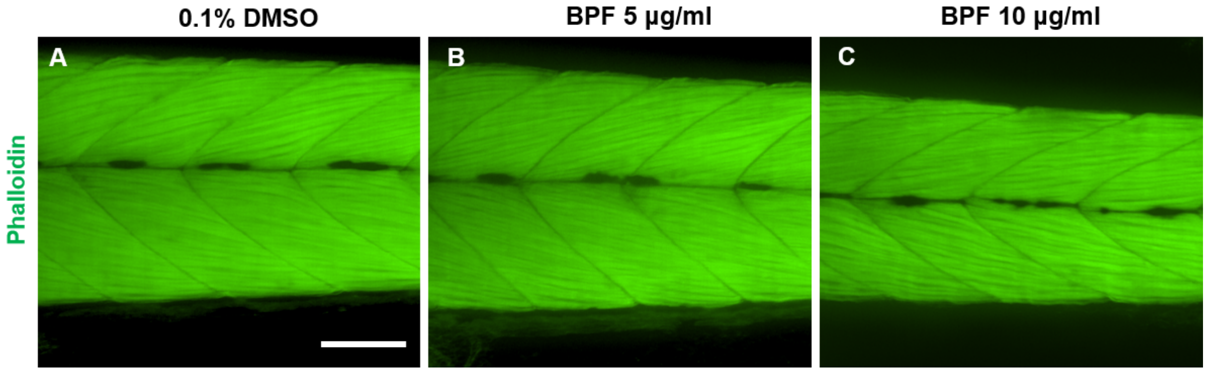

Figure S3. Effects of BPF on skeletal muscle of zebrafish larvae. (A–C) Lateral views of the trunk of zebrafish at 5 dpf. Labeling with Alexa Fluor 488 (green), a marker of α-actin. Scale bar, 100 μm. In BPF-exposed larvae, no difference was observed between muscles of larvae treated with BPF compared to untreated control larvae.
